# Naringenin Produces Neuroprotection Against LPS-Induced Dopamine Neurotoxicity via the Inhibition of Microglial NLRP3 Inflammasome Activation

**DOI:** 10.3389/fimmu.2019.00936

**Published:** 2019-05-01

**Authors:** Ce Chen, Yi-Zheng Wei, Xue-Mei He, Dai-Di Li, Guo-Qing Wang, Jing-Jie Li, Feng Zhang

**Affiliations:** Key Laboratory of Basic Pharmacology of Ministry of Education and Joint International Research Laboratory of Ethnomedicine of Ministry of Education, Zunyi Medical University, Zunyi, China

**Keywords:** Parkinson's disease, microglia, inflammasome, naringenin, neuroprotection

## Abstract

**Background:** Parkinson's disease (PD) is the second most prevalent central nervous system (CNS) degenerative disease and characterized by slow and progressive loss of dopamine (DA) neurons in the midbrain substantia nigra. Microglia-mediated neuroinflammation has been considered as the major central event in the process of DA neuronal loss. Thus, inhibition of neuroinflammation could possess a more viable strategy for PD treatment. Naringenin (NAR), a natural flavanoid contained in citrus fruit and grapefruits, possesses amounts of pharmacological activities. Recent studies indicated that NAR produced neuroprotection against several neurological disorders. However, the mechanisms underlying NAR-generated neuroprotection are not fully illuminated.

**Methods:** In the present study, rat nigral stereotaxic injection of lipopolysaccharide (LPS)-induced DA neuronal loss was performed to investigate NAR-mediated neuroprotection. In addition, BV-2 and MN9D cell lines were applied to explore the underlying mechanisms.

**Results:** NAR protected DA neurons against LPS-induced neurotoxicity. Also, NAR suppressed microglial nod-like receptor protein 3 (NLRP3) inflammasome signaling activation and the subsequent pro-inflammatory factors release. In addition, NAR-mediated DA neuroprotection was dependent on the inhibition of microglial NLRP3 inflammasome activation, as evidenced by the observations that NAR-reduced pro-inflammatory factors production and further NAR-exerted DA neuroprotection against LPS-induced neuronal damage was not discerned after microglial NLRP3 siRNA treatment.

**Conclusions:** This study demonstrated that NAR targeted microglial NLRP3 inflammasome to protect DA neurons against LPS-induced neurotoxicity. These findings suggest NAR might hold a promising therapeutic potential for PD.

## Background

Parkinson's disease (PD) is the second most prevalent central nervous system (CNS) degenerative disease. It is characterized by slow and progressive loss of dopamine (DA) neurons in the midbrain substantia nigra (SN) with the accumulation of α-synuclein in Lewy bodies and neuritis (1). Although the etiology of PD remains unclear, amounts of studies have suggested that neuroinflammation is recognized as the major central event in the process of DA neuronal cell death in PD (2). The hallmark of neuroinflammation is glial activation, especially microglia activation. Microglia are the chief innate immune cells within the CNS. Upon the introduction of stimuli, such as brain injury, inflammation and pathogens, microglia became activated and released a large number of neurotoxic factors, such as tumor necrosis factor-α (TNF-α), interleukin-1β (IL-1β) and IL-18, and then these factors would work in concert to trigger neurodegeneration (3).

Among these inflammatory factors, IL-1β was considered to be indispensable for the initiation and progress of DA neurodegeneration (4). Postmortem studies demonstrated that enhanced IL-1β expression was discerned in the microglia surrounding Lewy bodies in PD patients (5). It was known that the protease caspase-1 participated in inflammatory responses due to its essential role on modulating the cleavage of inactive precursor pro-IL-1avage in the cytosol (6). Furthermore, caspase-1 activity was regulated by cytosolic multi-protein complexes termed as inflammasome that consisted of the nod-like receptor protein (NLRP) family, adaptor protein ASC and pro-inflammatory precursor pro-caspase-1 (6). In addition, nod-like receptor protein 3 (NLRP3) inflammasome was highly located in microglia and involved in the process of neuroinflammation (7). Endogenous dangerous cues, such as bacterial toxins and urate crystals, extensively activated NLRP3 inflammasome (8). Activation of NLRP3 inflammasome has been demonstrated a significant contribution to the progression of neurodegenerative diseases (9, 10). Moreover, purinergic receptor (P2X7 receptor) blockers conferred neuroprotection against DA neurotoxicity through the inflammasome inactivation (11). Thus, inhibition of inflammasome activation might be developed to be a potential therapeutic intervention against PD.

At present, epidemiological studies have demonstrated that anti-inflammatory drugs were applied to lower the incidence risk of idiopathic PD (12). Given the potential role of neuroinflammation on the pathogenesis of PD, drug candidates with anti-inflammatory properties were greatly needed (13). Naringenin (NAR) is a natural flavanoid contained in citrus fruit and grapefruits. NAR has been revealed to possess multiple pharmacological properties, including anti-oxidative, anti-proliferative, anti-inflammatory and anti-tumor activities (14). The anti-inflammatory actions of NAR were well-confirmed (15). NAR protected against airway remodeling after mycoplasma pneumoniae infection via the inhibition of autophagy-mediated lung inflammation and fibrosis (16). Also, NAR inhibited the development of precancerous lesions through controlling inflammation in rat colon (17). Recently, there has been demonstrated that NAR could cross the blood–brain barrier (BBB) (18). Thus, growing interests have been attractive on its neuroprotection. NAR protected against PD animal models and attenuated neuroinflammatory reactions (19). However, the mechanisms underlying NAR-mediated neuroprotection and anti-neuroinflammatory effects remain unclarified. In the present study, we aimed to investigate whether NAR protected DA neurons via the inhibition of microglial NLRP3 inflammasome activation. First, we evaluated the neuroprotective effects of NAR on the LPS-induced neurotoxicity both *in vivo* and *in vitro*. In addition, BV-2 and MN9D cell lines were applied to explore the underlying mechanisms. These findings will give an insight into a promising potential therapeutic avenue for PD.

## Methods

### Reagents

Naringenin and lipopolysaccharide (LPS) were purchased from Sigma-Aldrich (St. Louis, MO, USA). MTT assay kit was obtained from Beijing Solarbio science and Technology Co., Ltd (Beijing, China). Enzyme-linked Immunosorbant assay (ELISA) kits for TNF-α, IL-1β, and IL-18 were bought from Elabscience Biotechnology Co., Ltd (Wuhan, China). Dulbecco's modified Eagle medium (DMEM) and OPTI-MEM were obtained from Gibco-BRL (Invitrogen Life Technologies, Carlsbad, USA). Anti-tyrosine hydroxylase (TH, Catalog No. Ab41528) and OX-42 (Catalog No. Ab1211) antibodies were purchased from Abcam (Cambridge, MA, USA). Anti-caspase-1 (Catalog. No. 22915-1-AP), ionized calcium-binding adapter molecule-1 (Iba-1, Catalog. No. 10904-1-AP), β-actin (Catalog. No. 20536-1-AP), Rabbit IgG (Catalog. No. SA00001-2), and mouse IgG (Catalog. No. SA00001-1) antibodies were purchased from Proteintech Group (Chicago, IL, USA). Anti-NLRP3 (Catalog. No. orb101128) antibody was purchased from Biorbyt (Cambridge, United Kingdom). Anti-ASC (Catalog. No. 67824) antibody was obtained from Cell Signaling Technology*, Inc*. (Beverly, MA, USA). The small interfering RNA (siRNA) against NLRP3 was purchased from GenePharma (Shanghai, China).

### Animal and Treatment

Adult male Sprague-Dawley rats (220–260 g) were purchased from the Experimental Animal Center of the Third Military Medical University (Chongqing, China; Specific pathogen-free Grade II; Certificate No. SCXK 2012-0011). All animal experiments were performed in accordance with Chinese Guidelines of Animal Care and Welfare and the present study was approved by the Animal Care and Use Committee of Zunyi Medical University (Zunyi, China). All the animals were housed in a temperature (25 ± 2°C) and humidity (60 ± 4%) environment, and given access to food and water *ad libitum*. Rats were acclimated to their environment for 1 week before the experiments. All the animals were randomly allocated to five experimental groups with six rats in each group: control (0.9% NaCl), NAR alone (100 mg/kg), LPS, LPS + NAR (50 mg/kg), and LPS + NAR (100 mg/kg). With anesthetized by 7% chloral hydrate (0.5 ml/100 g, v/w), rats received a single LPS (5 μg in 5 μl PBS) unilateral injection into the SN pars compacts followed by the coordinates 4.8 mm posterior to bregma, 1.7 mm lateral to the midline, and 8.2 mm ventral to the surface of the skull (20, 21). After seven daily intragastric administration of NAR, rat behavior was analyzed by rotarod test and then animals were sacrificed.

### Tissue Preparation and Immunohistochemistry

After rat motor performance was finished, rats were deeply anesthetized by 7% chloral hydrate, and transcardially perfused with cold PBS before 4% formaldehyde was used to fix. Then, brains were removed and post fixed with 4% formaldehyde for 48 h. Next, formaldehyde (4%) was replaced by 30% sucrose solution at 4°C until tissues sank. Brains were cut into 35-mm using a horizontal sliding microtome. A total of 36 consecutive brain slices through the entire SN was collected and used for the immunohistochemistry staining by every sixth section (22). DA neurons were recognized with an anti-TH antibody and microglia with OX-42. In brief, the brain slices were first treated with 0.3% Triton X-100 for 30 min and blocked with goat serum. Then, the slices were incubated overnight at 4°C with the following primary antibodies: TH (1: 800), OX-42 (1:800), and NLRP3 (1:800). Afterwards, brain tissues were incubated in fluorescent-conjugated secondary antibody for 1 h. Digital images of TH-positive neurons, OX-42-positive microglia and NLRP3-positive inflammasome in the SN were acquired by an Olympus microscope (Olympus, Tokyo, Japan) via an attached Polaroid digital microscope camera (Polaroid, Cambridge, MA, USA). Quantification of TH-positive neurons was determined by visually counting the number of TH-positive neuronal cell bodies. Activation of microglia and NLRP3 inflammasome was detected by the fluorescence intensity analysis of OX-42-positive microglia and NLRP3-positive inflammasome, respectively. The results were obtained from the average. The mean value was deduced by averaging the counts of six sections for each rat.

### Cell Culture and Treatment

BV-2 cells (microglia cell lines) were purchased from the Cell Culture Center in the Institute of Basic Medical Sciences of Chinese Academy of Medical Sciences (Wuhan, China). MN9D cells (DA neuron cell line) were purchased from the Cell Culture Center in the Institute of Basic Medical Sciences of Chinese Academy of Medical Sciences (Beijing, China). BV-2 cells were cultured in MEM medium with 10% fetal bovine serum (FBS) and 1% penicillin-streptomycin on an atmosphere with 5% CO_2_ at 37°C, while MN9D cells were cultured in DMEM medium. BV-2 cells were treated with different concentrations of NAR for 1 h followed by LPS treatment for an additional 24 h. Then, cells and culture medium were collected for the later indexes detection.

### Cell Viability Assay

Cells were cultured in 1 × 10^5^/well in 96-well plates for 24 h. Subsequently, the conditioned medium was replaced by a new medium with 2% FBS and then cells were exposed to LPS with or without NAR for another 24 h. Afterwards, 3-(4, 5-dimethylthiazol-2-yl)- 2,5-diphenyltetrazoliumbromide (MTT) was applied to determine cell viability. The absorbance was detected by an automated microplate (Sunnyvale, CA, USA) reader within 550 nm wavelength.

### ELISA

The levels of TNF-α, IL-1β, and IL-18 were measured by ELISA according to the manufacturer's instructions. The microplate reader was used to measure the absorbance at 450 nm.

### RNA Transfection

BV-2 cells were transfected with siRNA oligos against NLRP3. Cells were seeded on 24-well plates for 24 h. GP-siRNA-Mate plus was used to transfect with siRNAs. After 6 h of transfection, the transfection solution was removed and cells were rinsed with PBS and then replaced with MEM medium containing 2% FBS followed by treatment of NAR and/or LPS.

### Rotarod Test

Rotarod test was assessed by studying the muscular coordination with cylindrical arrangement of thin steel rods contained. First, rats were trained to adapt at a speed of 10 rpm/min before the experiment start. After intragastric administration of NAR for 7 days, the rods were divided into two parts with compartmentalization. With speed accelerating from 10 to 30 rpm over a period of 5 min, two rats were detected at the same time and the duration time each rat stayed on the rod was recorded and calculated for analyzing the behavior changes of rats.

### Western Blot Analysis

The total protein content was extracted from rat midbrain tissue and BV-2 cells using a lysis buffer containing protease inhibitors. The protein concentration was measured by the BCA protein assay. Equal amounts of protein were separated by sodium dodecyl sulfate/polyacrylamide gel electrophoresis and transferred to a polyvinylidene fluoride membrane. The membrane was blocked in TBS with Tween 20 containing 5% non-fat dry milk for 4 h and probed with primary antibodies overnight at 4°C and incubated with the following primary antibodies: TH (1:1,000), Iba-1 (1:800), NLRP3 (1:1,000), ASC (1:400), caspase-1 (1:800), β-actin (1: 2,000), and then incubated for 1 h with a horseradish-peroxidase-conjugated anti-mouse IgG antibody or anti-rabbit IgG at 1:2,000 dilution. Protein bands were visualized on X-ray film by utilizing an enhance chemiluminescence system before the enhanced ECL reagent used.

### Statistical Analysis

Data were indicated as mean ± standard error of the mean (SEM). Statistical significance was analyzed by one-way analysis of variance (ANOVA) using GraphPad Prism software (GraphPad Software Inc., San Diego, CA, USA). With ANOVA demonstrating the significant differences, pairwise comparison between means was evaluated by Bonferroni's *post-hoc test* with correction. A value of *p* < 0.05 was considered statistically significant.

## Results

### NAR Attenuated LPS-Induced DA Neuronal Loss *in vivo*

**Figure 1:**
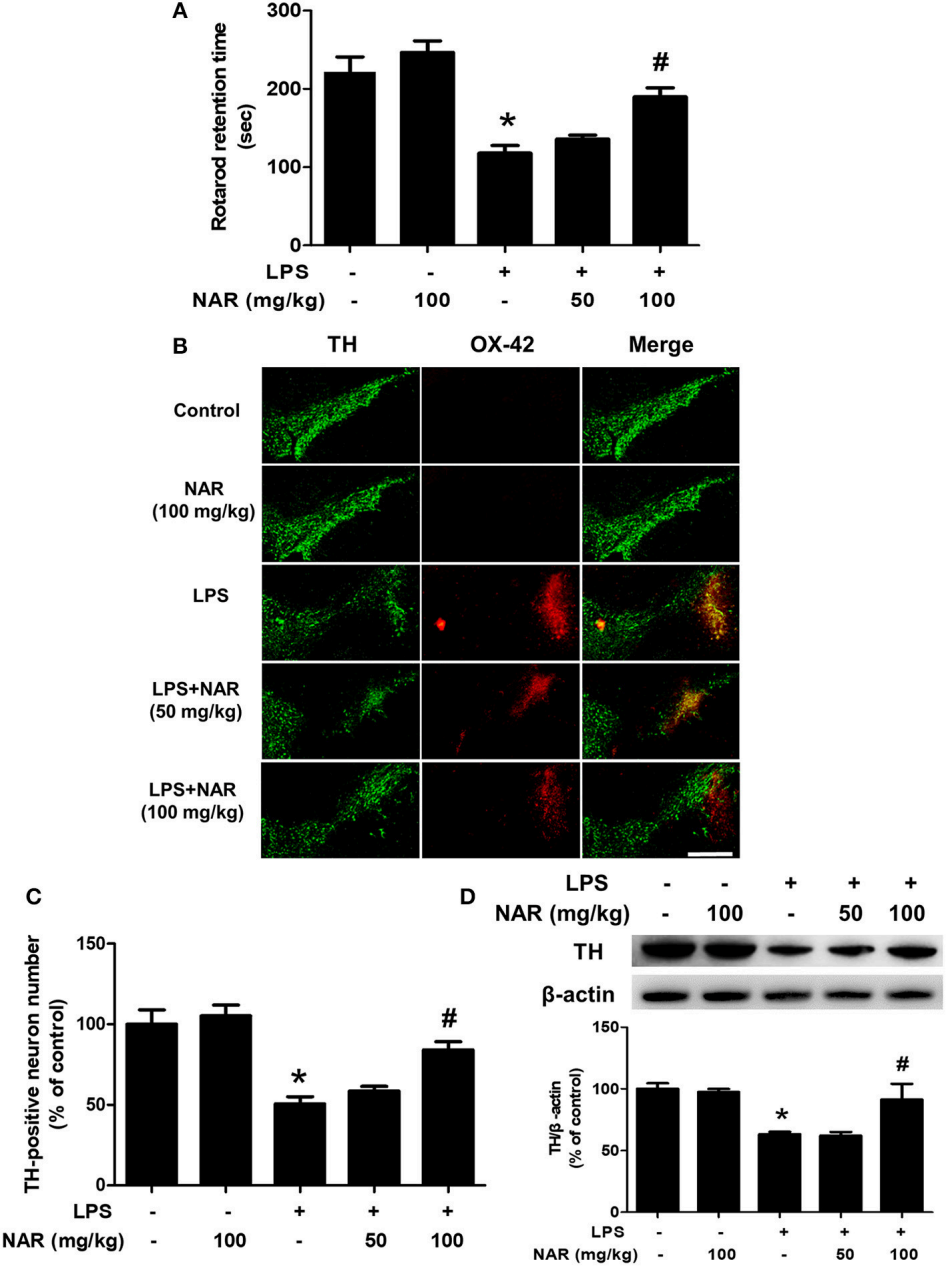
NAR attenuated LPS-induced DA neuronal loss *in vivo*. One day after the last treatment of NAR, rat behavior changes were evaluated by the rotarod test. The time rat stayed on the rod was recorded. **(A)** Then, rats were sacrificed and double-label immunofluorescence was performed by staining with anti-TH and anti-OX-42 antibodies (green fluorescence represented DA neurons and red fluorescence represented microglia. **(B)** Scale bar = 200 μm. The number of SN TH-positive neurons was counted **(C)** and TH protein expression was detected by western blot assay. **(D)** The ratio of densitometry values of TH with β-actin was analyzed and normalized to each respective control group. Data were expressed as a percentage of the control group and were the mean ± SEM from six rats. ^*^p < 0.05 compared with control group; ^#^*p* < 0.05 compared with LPS group.

After seven daily intragastric administration of NAR, rat behavior changes were analyzed by rotarod test. As shown in Figure 1A, LPS decreased the time rat stayed on the rod (54.86 ± 5.72%; *t* = 5.683, *p* < 0.05) and NAR attenuated LPS-induced decrease in the time rat remained on the rod (164.14 ± 8.48%; *t* = 3.935, *p* < 0.05). Results from DA neuronal counting and densitometric analysis as indicated in Figures 1B,C, LPS reduced DA neuronal loss compared with control group (52.64 ± 6.27%; *t* = 5.811, *p* < 0.05) and this reduction was inhibited by NAR treatment in a dose-dependent manner (170.62 ± 11.35%; *t* = 3.937, *p* < 0.05). To further confirm NAR-mediated DA neuroprotection, the effects of NAR on TH protein expression after LPS treatment were determined. As shown in Figure 1D, NAR ameliorated LPS-induced decrease in TH protein expression (145.93 ± 20.72%; *t* = 3.110, *p* < 0.05).

### NAR Inhibited the Activation of Microglia and NLRP3 Inflammasome *in vivo*

**Figure 2:**
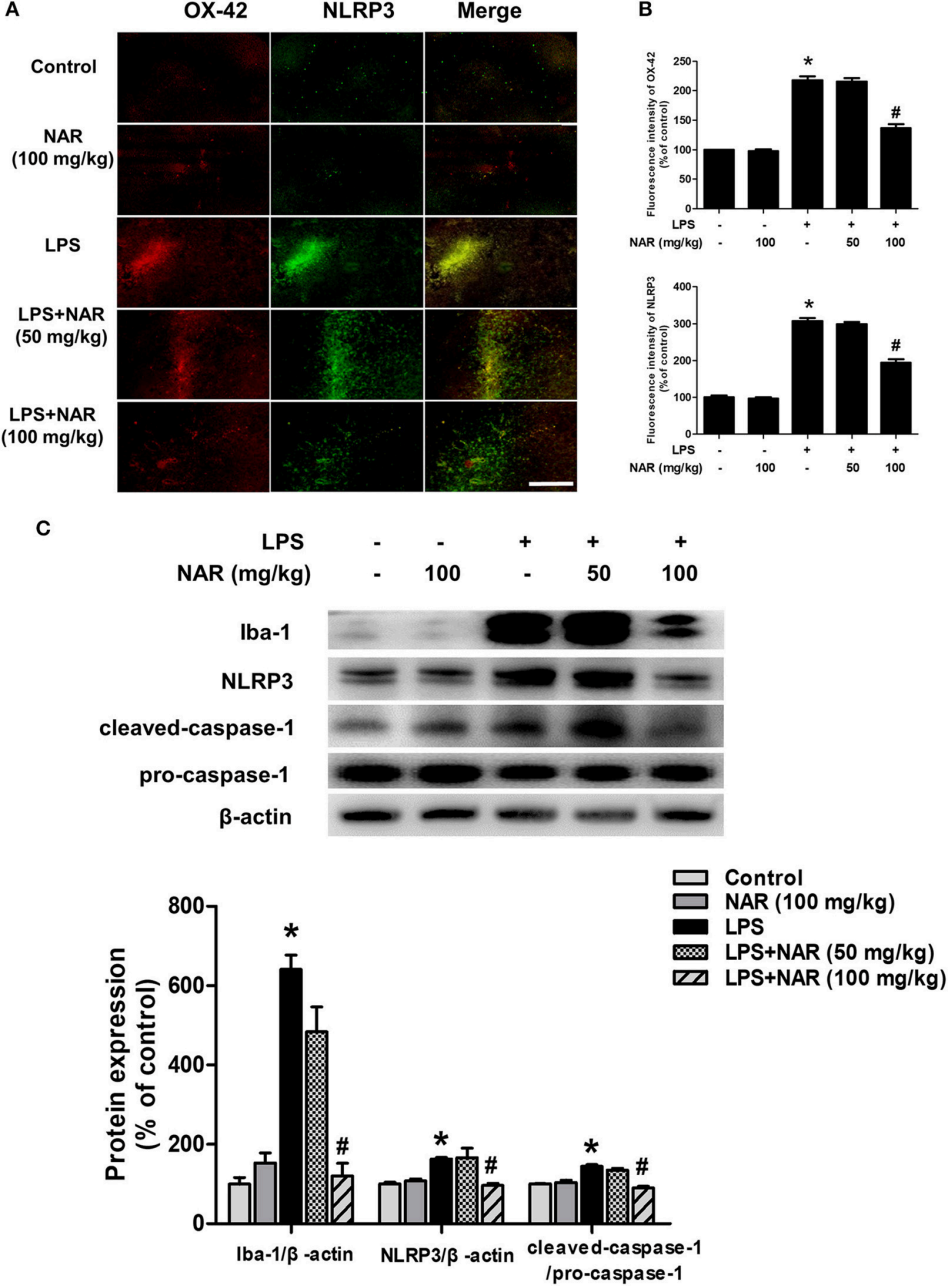
NAR inhibited microglial NLRP3 inflammasome activation *in vivo*. Rat brains were collected and stained by double-immunofluorescence with anti-NLRP3 and anti-OX-42 antibodies (green fluorescence represented NLRP3 inflammasome and red fluorescence represented microglia). **(A)** Scale bar = 200 μm. Quantification of the fluorescence intensity of the staining to assess OX42 and NLRP3 level. **(B)** The protein expressions of Iba-1, NLRP3, pro-caspase-1 and cleaved caspase-1 were detected by western blot assay. **(C)** The ratio of densitometry values of Iba-1 and NLRP3 with β-actin and cleaved caspase-1 with pro-caspase-1 was analyzed and normalized to each respective control group. Data were expressed as a percentage of the control group and were the mean ± SEM from six rats. ^*^p < 0.05 compared with control group; ^#^*p* < 0.05 compared with LPS group.

Next, we investigated the effects of NAR on microglia-induced neuroinflammation. Double-Immunofluorescence analysis was performed to determine the co-location of microglia and NLRP3 inflammasome. As shown in Figure 2A, in parallel with DA neuronal loss, LPS induced microglia activation and the activated state of microglia was suppressed by NAR. Since the activation of NLRP3 inflammasome was involved in the modulation of neuroinflammatory responses, the effects of NAR on LPS-elicited NLRP3 inflammasome activation were detected. As shown in Figure 2B, the activation of NLRP3 inflammasome was located in activated microglia and NAR could inhibit LPS-induced microglial (63.11 ± 3.78%; *t* = 11.01, *p* < 0.05) and NLRP3 inflammasome activation (63.41 ± 3.85%; *t* = 11.47, *p* < 0.05). Also, as shown in Figure 2C, NAR decreased LPS-induced protein expressions of Iba-1 (19.18 ± 5.59%; *t* = 7.982, *p* < 0.05), NLRP3 (59.36 ± 3.72%; *t* = 3.295, *p* < 0.05), and caspase-1 (62.78 ± 3.13%; *t* = 6.802, *p* < 0.05). As shown in Supplementary Figure 2, NAR decreased LPS-induced protein expressions of pro-IL-1β (*p* < 0.05) and IL-1β mRNA (*p* < 0.05).

### NAR Ameliorated LPS-Induced Microglial Activation and Pro-Inflammatory Factors Release *in vitro*

**Figure 3:**
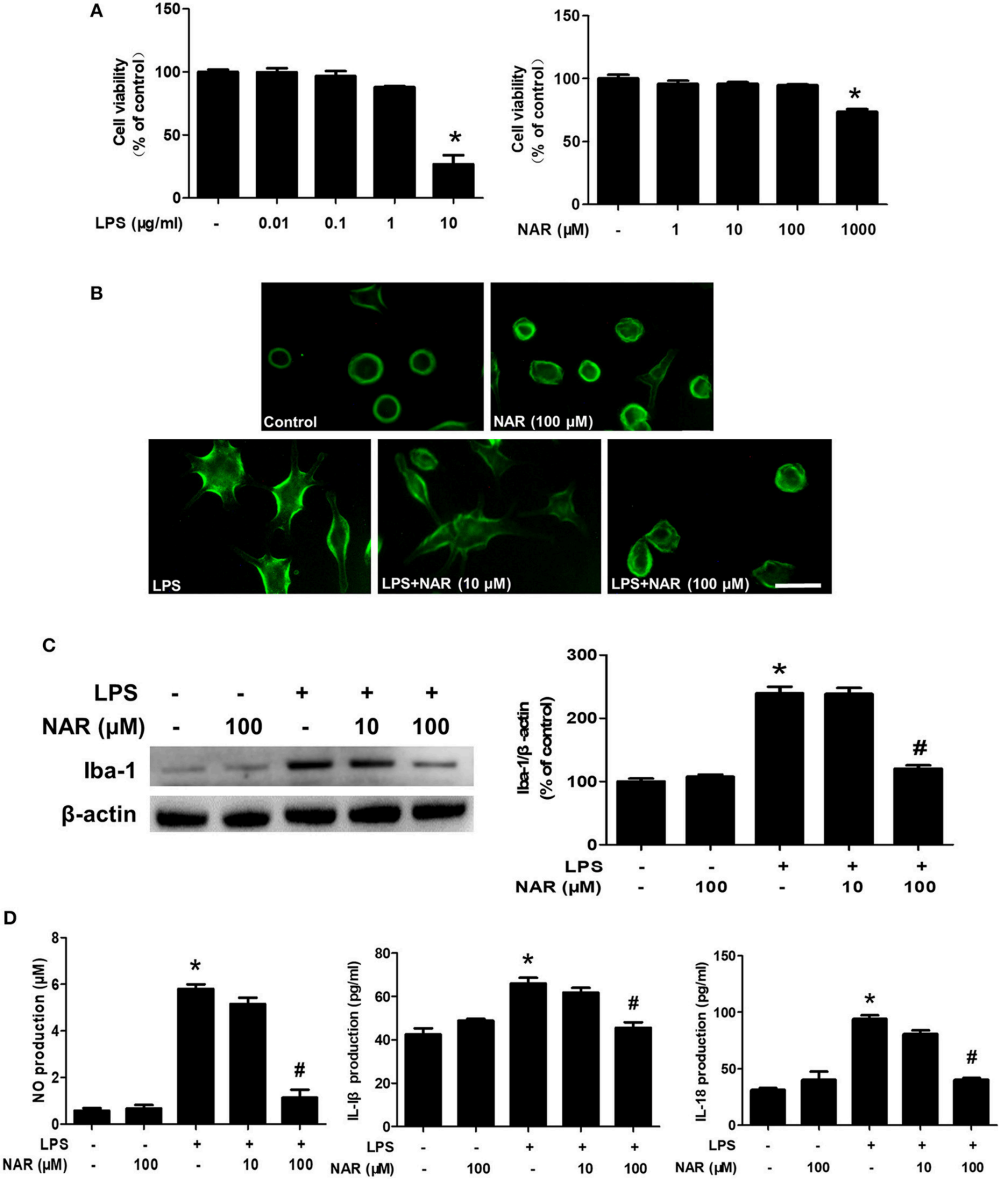
NAR ameliorated LPS-induced microglial activation and pro-inflammatory factors release *in vitro*. BV-2 cells were treated with different concentration of NAR and LPS for 24 h. Cell viability was determined by MTT assay. **(A)** Microglia activation was detected by immunofluorescence staining with an anti-Iba-1 antibody. **(B)** Scale bar = 100 μm. The level of Iba-1 protein expression in BV-2 cell was measured via western blot assay. **(C)** The ratio of densitometry values of Iba-1 with β-actin was assessed and normalized to each respective control cultures. The release of pro-inflammatory factors, such as IL-18, IL-1β, and NO, in the culture medium was detected by ELISA and Griess reagent, respectively. **(D)** Data were the mean ± SEM from three independent experiments performed in triplicate. ^*^p < 0.05 compared with control cultures; ^#^*p* < 0.05 compared with LPS-treated cultures.

To further confirm the inhibitory actions of NAR on microglia-elicited neuroinflammation, BV-2 cells were employed to examine the effects of NAR on LPS-induced microglial activation. First, MTT assay showed that LPS (0.01–1 μg/ml) and NAR (1–100 μM) had no significant cytotoxicity (Figure 3A). Second, microglia in LPS-treated cultures presented an enlarged cell body and irregular status from resting round and small cells in control cultures to the highly activated amoeboid shapes. NAR attenuated LPS-induced microglial activation (Figure 3B). Moreover, NAR suppressed LPS-induced Iba-1 protein expression (50.22 ± 0.28%; *t* = 11.75, *p* < 0.05) (Figure 3C). Then, the inhibitory ability of NAR on the production of microglial pro-inflammatory factors was assessed. As shown in Figure 3D, NAR reduced LPS-induced production of NO (19.61 ± 4.82%; *t* = 14.40, *p* < 0.05), IL-1β (69.27 ± 3.88%; *t* = 6.365, *p* < 0.05), and IL-18 (42.71 ± 2.15; *t* = 9.588, *p* < 0.05) in the culture medium.

### NAR Inhibited NLRP3 Inflammasome Activation Induced by LPS *in vitro*

**Figure 4:**
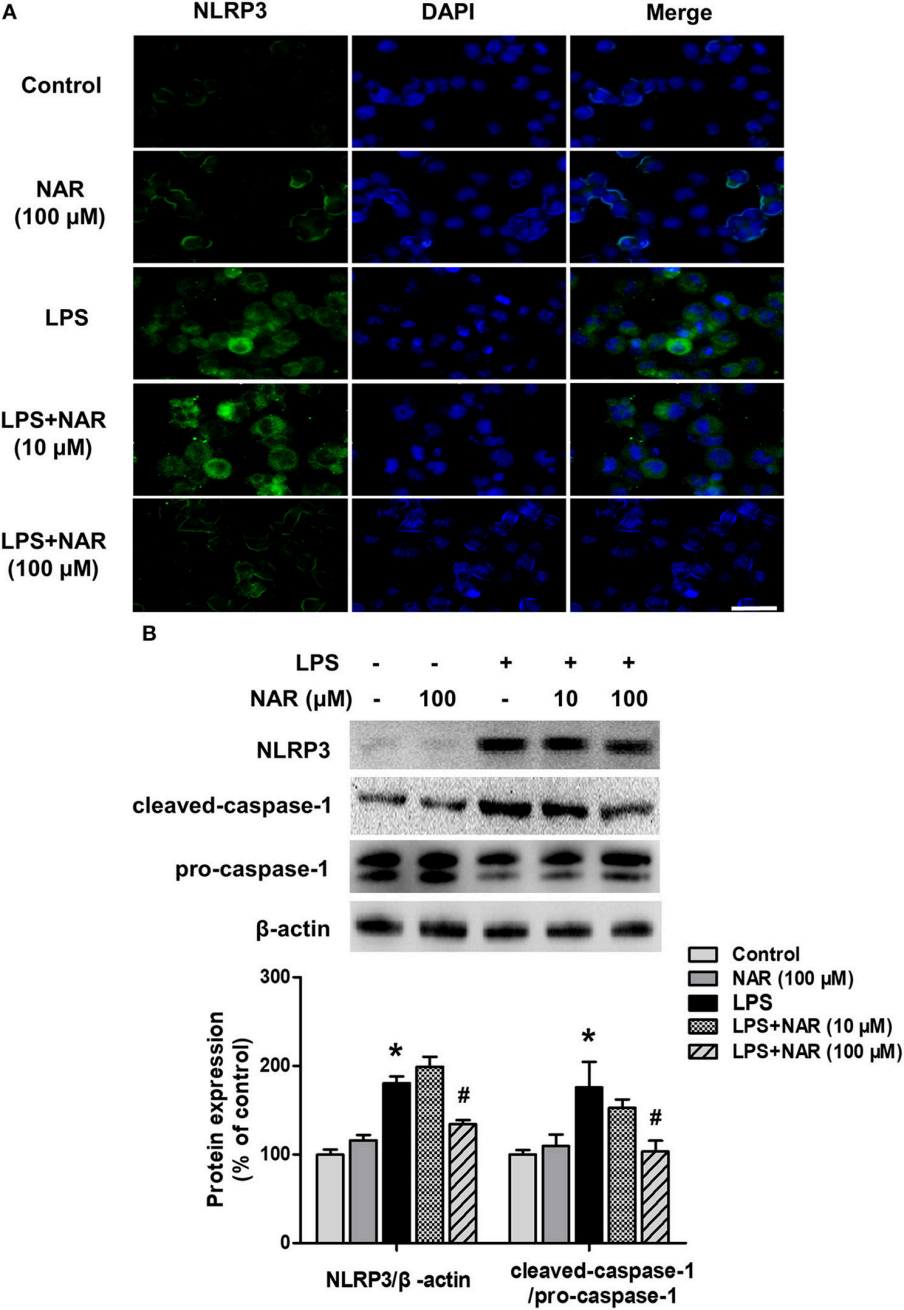
NAR inhibited LPS-induced activation of NLRP3 inflammasome *in vitro*. BV-2 cells were treated with NAR for 60 min followed by the application of LPS (1 μg/ml) for 24 h. Immunofluorescence detection of NLRP3 inflammasome in BV-2 cells were performed. **(A)** Scale bar = 100 μm. The protein expressions of NLRP3, cleaved caspase-1 and pro-caspase-1 were detected by western blot assay. **(B)** The ratio of densitometry values of NLRP3 with β-actin and cleaved caspase-1 with pro-caspase-1 was assessed and normalized to each respective control cultures. Data were the mean ± SEM from three independent experiments performed in triplicate. ^*^p < 0.05 compared with control cultures; ^#^*p* < 0.05 compared with LPS-treated cultures.

The effects of NAR on microglial NLRP3 inflammasome activation were further confirmed. As shown in Figure 4A, LPS induced NLRP3 inflammasome activation, and this activated state of NLRP3 inflammasome was attenuated by NAR treatment. Similarly, NAR inhibited LPS-induced activation of NLRP3 (74.58 ± 1.07%; *t* = 3.634, *p* < 0.05) and caspase-1 (62.97 ± 10.47%; *t* = 2.647, *p* < 0.05) (Figure 4B).

### NLRP3 Inflammasome Participated in NAR-Mediated Anti-Inflammatory Actions

**Figure 5:**
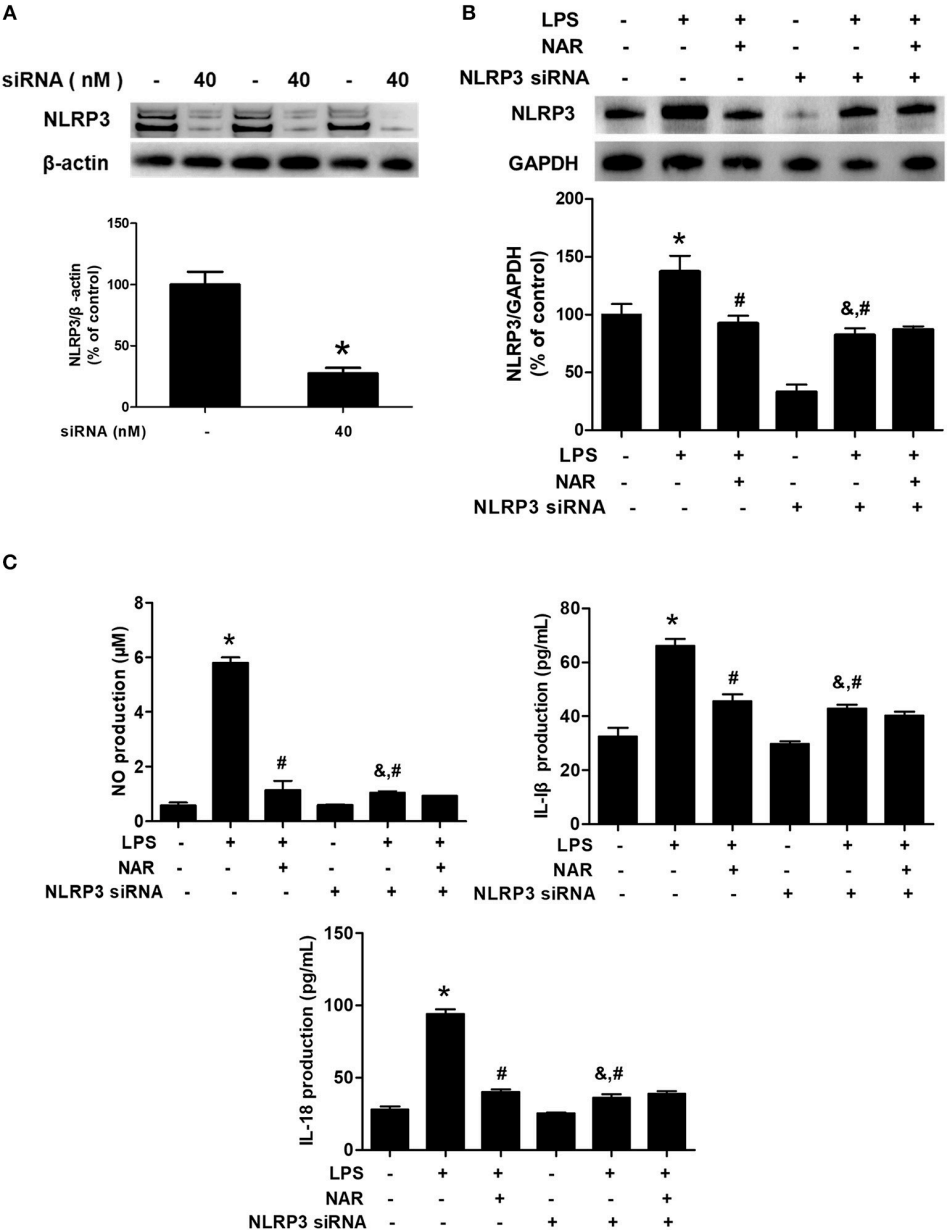
NLRP3 inflammasome participated in NAR-mediated anti-inflammatory actions. BV-2 cells were treated with NLRP3 siRNA (40 nmol/L). After 6 h of transfection, the transfection solution was removed and cells were rinsed with PBS. The silence efficiency was validated by western blotting. **(A)** Then, cells were treated with NAR (100 μM) for 60 min and stimulated by LPS (1 μg/ml) for 24 h. The NLRP3 protein expression in BV-2 cells were detected via western blot assay. **(B)** The production of IL-18, IL-1β, and NO in the culture medium was detected by ELISA and Griess regent, respectively. **(C)** Data were the mean ± SEM from three independent experiments performed in triplicate. ^*^p < 0.05 compared with control cultures; ^#^*p* < 0.05 compared with LPS-treated cultures. ^&^*p* < 0.05 compared with NLRP3 siRNA alone-treated cultures.

To investigate the role of NLRP3 inflammasome on NAR-mediated anti-inflammatory properties, NLRP3 siRNA (Nlrp3-Mus-727 siRNA oligo) was applied in the cell cultures. The successful transfection with NLRP3 siRNA was verified by NLRP3 protein level in cells (28.09 ± 4.84%, *p* < 0.05) (Figure 5A). Meanwhile, as shown in Figure 5B, LPS still induced NLRP3 activation after NLRP3 siRNA treatment compared with cells only treated with NLRP3 siRNA (264.03 ± 31.40%; *t* = 4.269, *p* < 0.05) but the lower NLRP3 activation was shown in LPS+NLRP3 siRNA than that in LPS alone treatment cultures (60.70 ± 7.23%; *t* = 4.741, *p* < 0.05). However, no significant difference of NLRP3 protein expression between LPS+NLRP3 siRNA and LPS+NAR+NLRP3 siRNA was detected. Furthermore, the effects of NAR on pro-inflammatory factors release after NLRP3 siRNA treatment were determined. As shown in Figure 5C, LPS also increased the production of NO (177.75 ± 13.48%; *t* = 1.906%, *p* < 0.05), IL-1β (144.18 ± 7.80%; *t* = 4.200, *p* < 0.05), and IL-18 (144.22 ± 7.58%; *t* = 3.733, *p* < 0.05) after NLRP3 siRNA treatment but this increase was not inhibited after NAR administration. Collectively, these results suggested that NLRP3 inflammasome was involved in NAR-exerted anti-neuroinflammatory actions.

### NAR Targeted Microglial NLRP3 Inflammasome to Protect DA Neurons Against LPS-Induced Neurotoxicity

**Figure 6:**
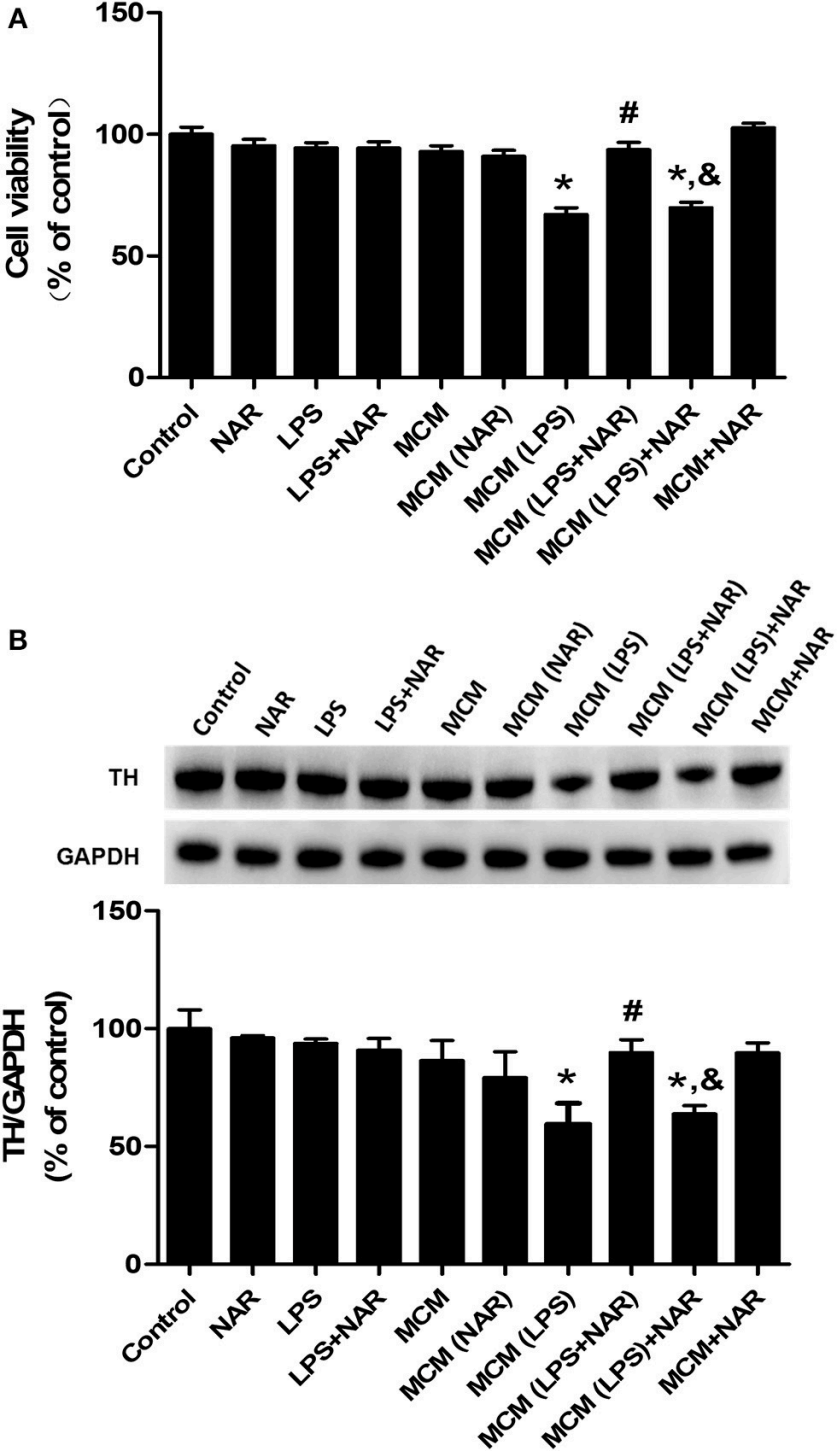
NAR protected DA neurons through the attenuation of microglial activation. Microglia-conditioned medium (MCM) was first prepared from BV-2 cell cultures. BV-2 cells were pretreated with NAR (100 μM) for 1 h followed by LPS (1 μg/ml) stimulation for 24 h. Then, MCM without any treatment, MCM with NAR alone treatment [MCM (NAR)], MCM with LPS treatment [MCM (LPS)], MCM with LPS and NAR treatment [MCM (LPS + NAR)] were collected and dialyzed and added back to MN9D cells followed by the incubation for additional 24 h. Then, MN9D cell viability was determined by MTT assay. **(A)** TH protein expression in MN9D cell was detected via western blot assay. **(B)** Data were the mean ± SEM from three independent experiments performed in triplicate. ^*^p < 0.05 compared with control cultures; ^#^*p* < 0.05 compared with MCM (LPS)-treated cultures. ^&^*p* < 0.05 compared with MCM (LPS+NAR)-treated cultures.

To further observe the link of inhibition of microglial activation with NAR-mediated DA neuroprotection, microglia-conditioned medium (MCM) was prepared from BV-2 cell cultures and added back to MN9D cell cultures. First, cell viability assay (Figure 6A) indicated that direct application of LPS on MN9D cells had no significant neurotoxicity, whereas MCM (LPS) caused neuronal damage (67.10 ± 1.78 %; *t* = 9.251, *p* < 0.05). Additionally, compared with MCM (LPS) group, MCM (LPS+NAR) produced neuroprotection from MCM (LPS)-induced neurotoxicity (139.92 ± 3.62%; *t* = 7.482, *p* < 0.05). However, with MN9D cells treated with MCM (LPS) followed by NAR treatment, no neuroprotection was exhibited. Results from TH protein expression detection were consistent with cell viability assay (Figure 6B), which together implied that NAR-mediated DA neuroprotection was microglia-dependent.

**Figure 7:**
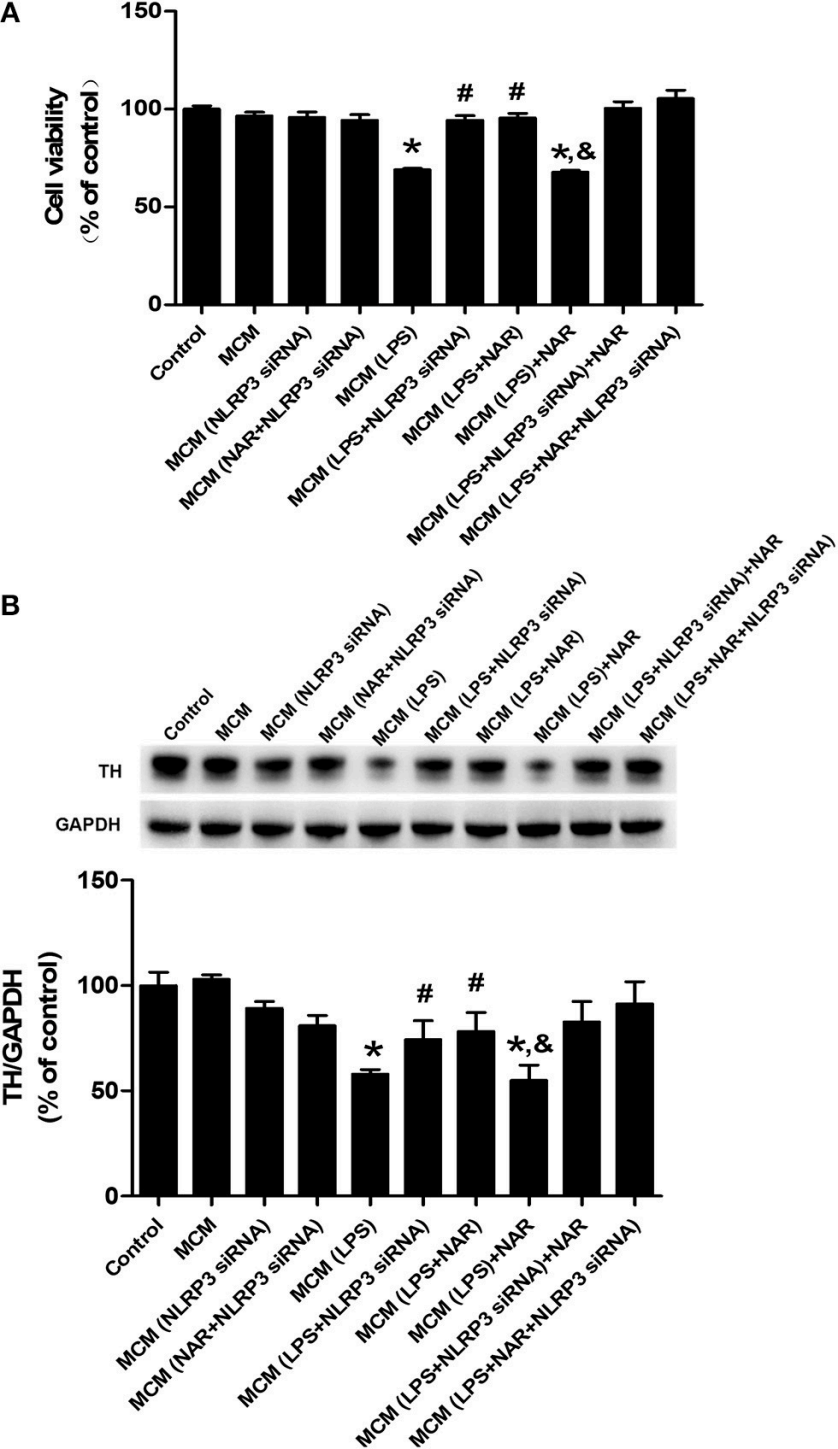
Microglial NLRP3 inflammasome was involved in NAR-conferred DA neuroprotection. BV-2 cell cultures were transfected with NLRP3-siRNA for 6 h. Then, cells were treated with NAR (100 μM) for 1 h followed by LPS (1 μg/ml) stimulation for 24 h. The microglia-conditioned medium (MCM) without any treatment, MCM with NAR alone treatment [MCM (NAR)], MCM with NLRP3 siRNA treatment [MCM (NLRP3 siRNA), MCM with LPS treatment (MCM (LPS)], MCM with LPS and NAR treatment [MCM (LPS + NAR)], MCM with LPS + NLRP3 siRNA treatment [MCM (LPS + NLRP3 siRNA)], MCM with NAR + NLRP3 siRNA treatment [MCM (NAR + NLRP3 siRNA)], MCM with LPS + NAR + NLRP3 siRNA treatment [MCM (LPS+NAR + NLRP3 siRNA)] were harvested and added to MN9D cells incubation for 24 h. Finally, MN9D cell viability was determined by MTT assay. **(A)** TH protein level in MN9D cells was determined by western blotting. **(B)** Data were the mean ± SEM from three independent experiments performed in triplicate. ^*^p < 0.05 compared with control cultures; ^#^*p* < 0.05 compared with MCM (LPS)-treated cultures. ^&^*p* < 0.05 compared with MCM (LPS + NAR)-treated cultures.

Since microglia were the target of NAR-produced neuroprotection, we next examined whether NAR-mediated DA neuroprotection was attributable to inhibition of microglial NLRP3 inflammasome activation. As shown in Figure 7A, compared with MCM (LPS) group, MCM (LPS+NLRP3 siRNA) (136.79 ± 3.73%; *t* = 8.852, *p* < 0.05) and MCM (LPS+NAR) (138.42 ± 3.43%; *t* = 7.474, *p* < 0.05) protected against MCM (LPS)-induced neurotoxicity, while no significant difference of cell viability between these two groups was indicated. Compared with MCM (LPS + NLRP3 siRNA) and MCM (LPS + NAR) groups, MCM (LPS + NAR+NLRP3 siRNA) didn't produce more neuroprotection from MCM (LPS)-elicited neuronal damage. Similar observation was demonstrated in TH protein expression assessment (Figure 7B).

## Discussion

This study presented that NAR protected DA neurons against LPS-induced neurotoxicity. Moreover, NAR suppressed microglial activation and the subsequent neuroinflammatory factors release. In addition, NAR-mediated DA neuroprotection was dependent on the inhibition of microglial NLRP3 inflammasome signaling activaton, as evidenced by the observations that NAR-reduced pro-inflammatory factors production and further NAR-exerted DA neuroprotection against LPS-induced neuronal damage was not discerned after microglial NLRP3 siRNA treatment. These findings suggested NAR-mediated DA neuroprotection might be attributable to the inhibition of microglia-induced neuroinflammation via NLRP3 inflammasome inactivation (Graphical Abstract).

**Graphical Abstract:**
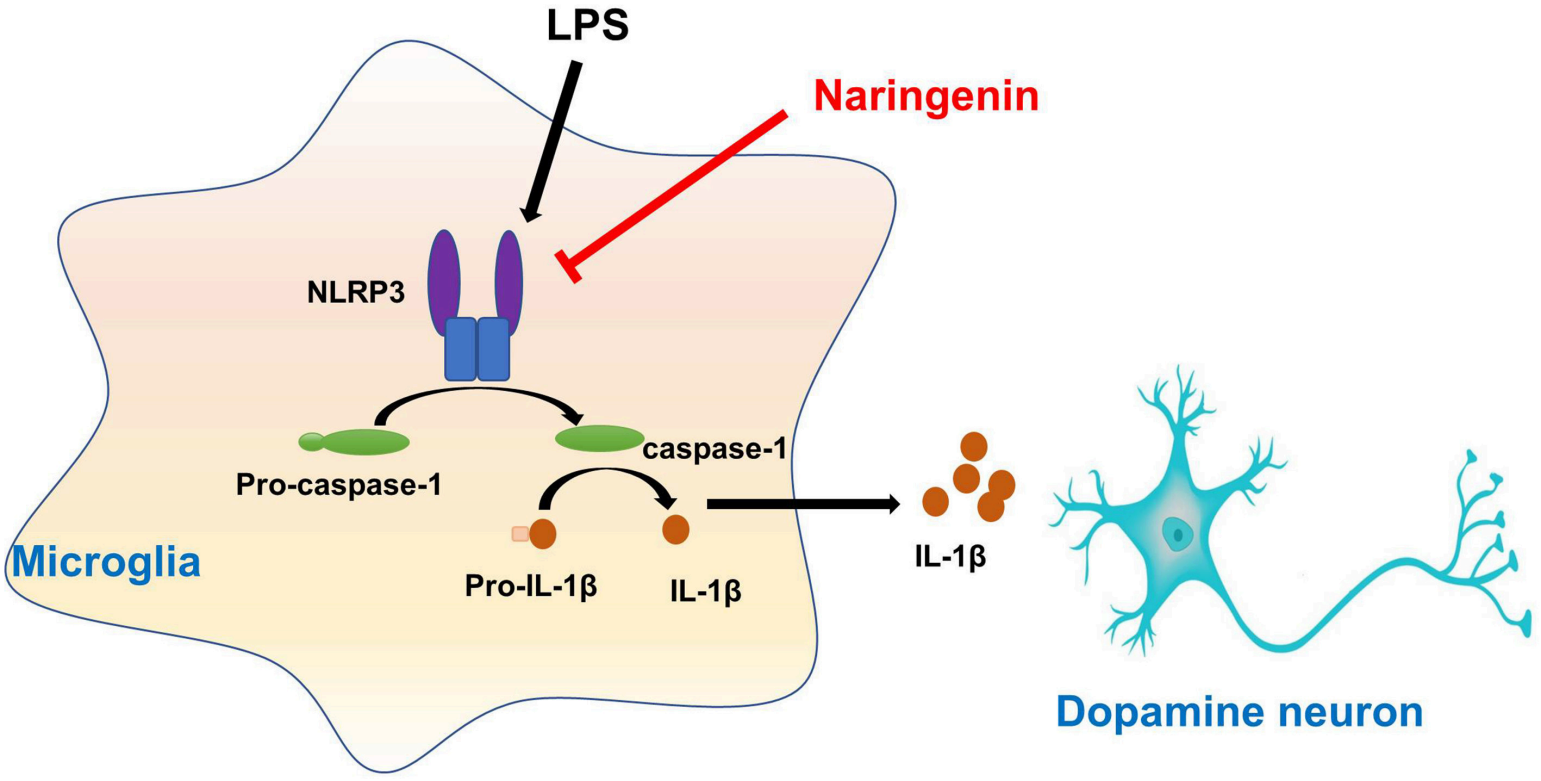
Naringenin produces neuroprotection via the inhibition of microglial NLRP3 inflammasome activation.

Neuroinflammation is a well-characterized pathophysiology occurring in close relation to the progression of PD (23). Understanding the cellular and molecular basis of neuroinflammation is essential for elucidating its impact on the incidence and progression of PD and other neurodegenerative diseases (24). Microglia play a pivotal role in regulation of immune response and neuronal homeostasis. Once activated by brain injury and inflammogen, microglia initiate an inflammatory cascade and release amounts of inflammatory cytokines and neurotoxic mediators. The accumulation of these factors augments DA neurodegeneration and thus DA neuronal loss. However, the continuing dying/dead DA neurons secrete several kinds of toxic substances, such as α-synuclein and danger-associated molecular patterns (DAMPs). These substances in turn lead to the secondary activation of microglia and the activated microglia further result in DA neuronal damage (25). Taken together, a vicious cycle creating the prolonged neuroinflammation and the progressive DA neurodegeneration was emerged (26). Therefore, the inhibition of microglia-mediated neuroinflammation is becoming a promising therapeutic strategy for PD treatment. Here, the present study found NAR protected DA neurons against LPS-induced neurotoxicity and reduced microglial activation and pro-inflammatory factors production. These findings suggested that inhibition of microglial activation and the pro-inflammatory factors sceretion participated in NAR-exerted DA neuroprotection.

In addition, inflammasomes are identified as intracellular pro-inflammatory pattern-recognition receptors able to initiate and propagate neuroinflammation. These cellular multiple-proteins are well-characterized in the innate immune system and activity of the NLRP3 inflammasome in CNS has been confirmed in microglia (27). We also detected the level of NLRP3 protein expression in rat primary astrocyte. Results indicated that NLRP3 was rarely expressed in astrocyte shown in Supplementary Figure 1. NLRP3 inflammasome activity is verified to be associated with neurodegenerative diseases (28). Also, NLRP3 inflammasome is indispensable for neuroinflammation and nigral DA neuronal loss in PD patients and animal models. Several lines of evidence indicated that mice lacking either NLRP3 or the key inflammasome effector caspase 1 were resistant to nigral DA neuronal loss resulting from exposure to pesticide (rotenone) and neurotoxin [1-Methyl-4-phenyl-1,2,3,6-tetrahydropyridine (MPTP)] (29, 30). Recent studies supported that the pathogenic NLRP3/capase-1/IL-1β axis played a critical role for in 6-hydroxydopamine (6-OHDA)-induced PD rat model (31). Besides, lysosome destabilization and cellular stress, such as potassium efflux and reactive oxygen species (ROS) generation, have been demonstrated to serves as a “bridging” mechanism of microbial proteins-elicited NLRP3 inflammasome activation (32). Furtherly, pathologic α-synuclein in cultured glia and mitochondrial ROS in monocytes triggered the activation of NLRP3 inflammasome during the progression of both the idiopathic and monogenic forms of PD (33, 34). It is noteworthy that DA neurons might offer a functional microenvironment where inflammasomes could be activated to initiate and propagate neuroinflammation although not yet characterized in PD patients. Thus, illuminating the role of NLRP3 inflammasome activity in DA neurons in response to PD-associated sterile inflammatory reactions would be of broad interest, as well as understanding how DA neurons initiated chronic neuroinflammation in PD would shed light on the underlying mechanisms upon the earliest stages of PD progression. In the present study, NAR-mediated neuroprotection was attributed to the inhibition of microglial NLRP3 inflammasome activation. This fact was supported by the following observations: 1) NAR inhibited microglial NLRP3 and pro-caspase-1 activation and the subsequent IL-1β and IL-18 production. 2) NAR could not further reduce LPS-induced inflammatory factors release and produce DA neuroprotection after NLRP3 siRNA administration. Together, this study demonstrated that NAR suppressed microglial NLRP3 inflammasome activation and reduced inflammatory factors secretion, and thus conferring DA neuroprotection. These findings supported that inhibition of NLRP3 inflammasome is considered as a potential therapeutic target for neurodegenerative diseases.

At present, current available drugs are focused on alleviating the symptoms of PD but are inadequate for halting the progressive PD process. However, the severe side-effects of the available drugs hold huge challenges for long-term therapy. Therefore, the more effective therapeutic agents and strategies are urgently required to delay or stop the progression of PD. Current evidence indicated that neuroinflammation could accelerate PD progression and inhibition of neuroinflammation would attenuate DA neuronal loss in PD. Thus, treatment with anti-inflammatory agents might be an important breakpoint for the pharmacotherapy of PD. However, the low success rate of translating potential anti-inflammatory candidates from animal studies to clinical trials was emerged. Therefore, an urgent need for better anti-inflammatory alternatives design was prompted. The present study revealed that NAR produced DA neuroprotection via inhibiting microglial NLRP3 activation and further neuroinflammatory responses both *in vivo* and *in vitro*. However, these findings resulted from experimental animal models and thus warrant rigorous investigation in human studies.

## Conclusions

This study demonstrated that NAR produced DA neuroprotection from LPS-induced neurotoxicity via the inhibition of microglial NLRP3 inflammasome activation. These findings suggest NAR might hold a promising therapeutic potential for PD.

## Ethics Statement

All animal experiments were performed in accordance with Chinese Guidelines of Animal Care and Welfare, and the present study was approved by the Animal Care and Use Committee of Zunyi Medical University (Zunyi, China).

## Author Contributions

FZ conceived and designed the experiments. All the authors participated in the experiments performance and data analysis. FZ wrote, revised, and checked the manuscript. All authors read and revised and approved the final manuscript.

### Conflict of Interest Statement

The authors declare that the research was conducted in the absence of any commercial or financial relationships that could be construed as a potential conflict of interest.

## Supplementary Material

Supplementary Figure 1 Primary astroglia cultures were treated with NAR for 60 min followed by the application of LPS (1 μg/ml) for 24 h. The level of NLRP3 protein expression in primary astroglia was measured via western blot assay. Data were the mean ± SEM from three independent experiments performed in triplicate. ^*^*p* < 0.05 compared with control cultures; #*p* < 0.05 compared with LPS-treated cultures.

Supplementary Figure 2 Rat brains were collected and the level of pro-IL-1β and IL-1β protein expression was measured via western blot assay **(A)**. Next, real time RT-PCR was used to investigate the gene expression of the pro-IL-1β and IL-1β **(B)**. Data were the mean ± SEM from 6 rats. ^*^*p* < 0.05 compared with control group; #*p* < 0.05 compared with LPS-treated group.
