# Supplementary figures and images for: Naringenin Produces Neuroprotection Against LPS-Induced Dopamine Neurotoxicity via the Inhibition of Microglial NLRP3 Inflammasome Activation
Source: Front Immunol. 2019 May 1;10:936. doi: 10.3389/fimmu.2019.00936 (PMC6504827; doi:10.3389/fimmu.2019.00936)

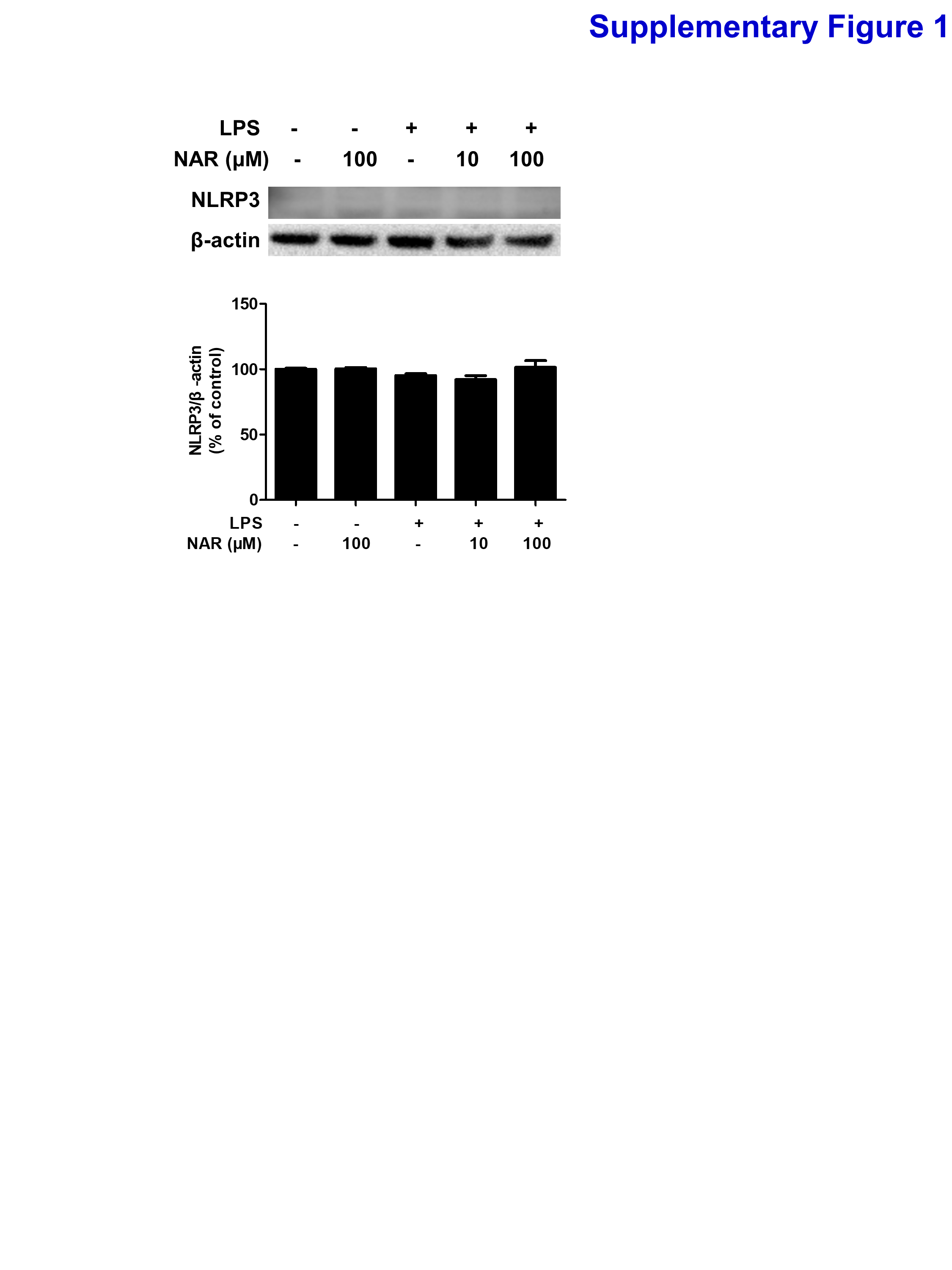

Supplement: Supplementary Figure 1 — Primary astroglia cultures were treated with NAR for 60 min followed by the application of LPS (1 μg/ml) for 24 h. The level of NLRP3 protein expression in primary astroglia was measured via western blot assay. Data were the mean ± SEM from three independent experiments performed in triplicate. *p < 0.05 compared with control cultures; #p < 0.05 compared with LPS-treated cultures. [file Image_1.tif]

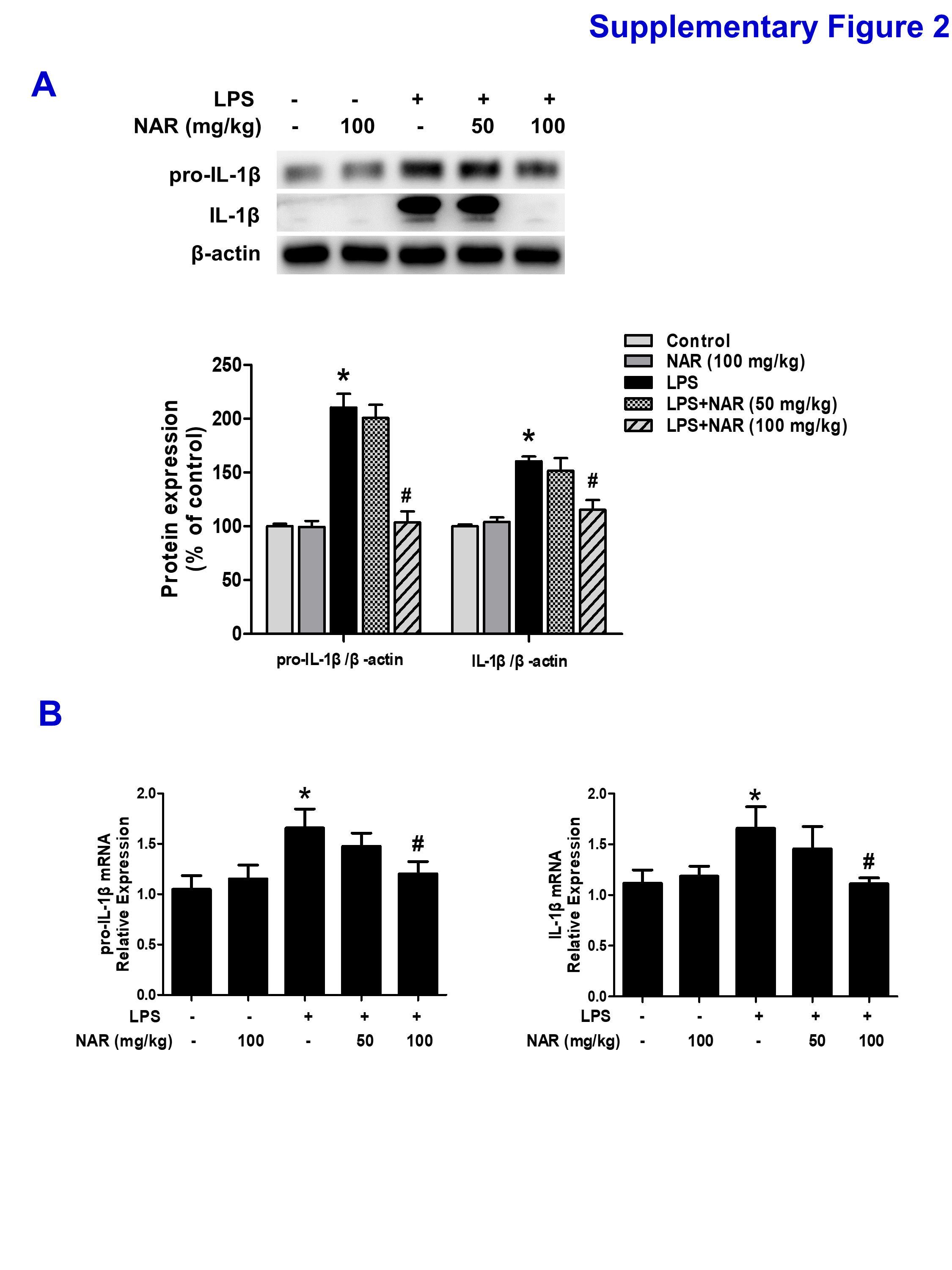

Supplement: Supplementary Figure 2 — Rat brains were collected and the level of pro-IL-1β and IL-1β protein expression was measured via western blot assay (A). Next, real time RT-PCR was used to investigate the gene expression of the pro-IL-1β and IL-1β (B). Data were the mean ± SEM from 6 rats. *p < 0.05 compared with control group; #p < 0.05 compared with LPS-treated group. [file Image_2.tif]
